# A community-led mobile health clinic to improve structural and social determinants of health among (im)migrant workers

**DOI:** 10.1186/s12939-022-01630-7

**Published:** 2022-05-02

**Authors:** Shannon Guillot-Wright, N. Miles Farr, Ellie Cherryhomes

**Affiliations:** 1 Department of Family Medicine; Center for Violence Prevention, University of Texas Medical Branch, Galveston, TX USA; 2 Internal Medicine; Community Engagement and Education, University of Texas Medical Branch, Galveston, TX USA

**Keywords:** Health equity, Structural violence, Migrant workers, Social determinants of health, Community-based participatory research, Mobile clinic

## Abstract

**Background:**

Community-led interventions that address structural and social determinants of health are lacking among (im)migrant workers, especially seafood workers. This lack of medical attention is especially alarming given their high rate of injury and death.

**Methods:**

Community-based participatory research (CBPR), a relational model that values the participants as equal partners in research, dissemination, and implementation, guided the interviews and mobile clinic. Seafood workers were engaged throughout data collection, analysis, and interpretation and played a significant role in moving the findings from research into actionable change.

**Results:**

To address the lack of healthcare options for (im)migrants, and at the request of the seafood workers participating in the ongoing CBPR study, we successfully implemented and treated workers in our mobile clinic.

**Discussion:**

Many of these individuals had not been seen by a healthcare provider in years, highlighting the importance of community trust and rapport building when addressing interconnected health and safety issues.

**Conclusions:**

Although CBPR and free (mobile) health clinics are in and of themselves not novel concepts, when applied to high-risk occupational settings with under-reached populations (e.g., (im)migrant workers), they have the ability to improve health and prevent injury. This intervention adds to the growing literature detailing the potential benefits of using CBPR, and meeting people where they are, especially with historically marginalized populations.

## Background

With a fatality rate 29 times higher than the national average, commercial fishing is one of the most dangerous industries in the US; especially true for the Gulf of Mexico, which ranks as one of the most vulnerable regions to experience falls overboard [1]. The volatile and hidden nature of their work creates inequitable work arrangements that result in a host of adverse mental and physical health disparities, including increased risk of workplace injuries, limited access to safe and secure employment, and heightened vulnerability to disease and death [2, 3]. Increased attention to injury, illness, and death among maritime workers, and commercial fishermen in particular, has resulted in improved mortality and morbidity rates. However, commercial fishing remains the most dangerous occupation in the US and includes some of the most vulnerable workers, including low-wage, aging, and (im)migrant workers [1, 4]. Histories of structural violence, such as racism and classism, combined with some of the nation’s harshest working conditions, further contribute to their health inequities [5]. Therefore, community-led interventions that address structural and social determinants of health should be implemented among (im)migrant workers, especially seafood workers.

To address this gap in need, our team interviewed fishermen to understand their health needs and then implemented a free mobile health clinic at the docks based on the interview results. Interviews were funded by the Southwest Center for Agricultural Health, Injury Prevention, and Education through an ongoing community-based participatory research (CBPR) study and the health clinic was partially funded through a partnership with the National Institute for Occupational Safety and Health (NIOSH) to increase COVID-19 vaccination outreach among Gulf Coast workers, specifically those in the seafood industry. (Im)migrant shrimp fishermen requested that the free health clinic be situated at the docks and we responded to this community-driven request by coordinating COVID-19 vaccines alongside other health and social services. The goals of the mobile health clinic were to 1) provide accessible health and social services for a population who cannot or will not visit traditional health clinics, 2) address socioeconomic needs to prevent future medical conditions, and 3) reduce COVID-19 transmission among a group who live in close quarters.

## Methods

### Procedure

The CBPR project was implemented in two phases. In phase 1, we interviewed shrimp fishermen at a southeast Texas dock and in Phase 2, we interviewed shrimp fishermen at a dock in the Texas Rio Grande Valley. Both phases were concentrated on reducing fatal and non-fatal injury among Gulf Coast fishermen based on feedback from the workers. For this paper, we only discuss the implementation results from Phase 1. Our team used CBPR methods because it is a relational model that values the participants as equal partners in research, dissemination, and implementation [6]. At the process level, communities are engaged throughout data collection, analysis, and interpretation. In the outcome phase, they play a significant role in moving the findings from research into action for change. CBPR can mitigate barriers of medical mistrust when participants are co-collaborators and increase long-term project sustainability [7]. The study was approved by the lead author’s Institutional Review Board.

### Participants and sampling

Interviews (*n* = 46) and observations (53 h) were conducted with shrimpers along the Gulf Coast. Upon receiving approval from company managers, recruitment occurred in the context of relationship building at docks throughout southeast Texas. A trusting environment was created by sitting at the docks 2-3 times a week for 1-2 h and waiting for fishermen to approach us. In total, the research team spent over 50 h at the docks for over 6 months. Initially, the manager identified shrimpers and then snowball sampling was used. For example, the dock manager identified shrimpers who lived at the docks and gave our team permission to be there early in the morning when they were more likely to be available. Subsequently, other fishermen came to know us through these meetings and agreed to be part of the research project. Interviewees understood their jobs or wages would not be impacted by participation and each received a $25 incentive for participating. A total of 46 (im)migrant Gulf Coast shrimpers were in Phase 1 of the CBPR project. The participants were primarily male (98%) and either Vietnamese (87%) or Latinx (13%). Approximately 30% of the participants had a history of homelessness or unstable housing. Citizenship status was purposefully not collected. The collection of citizenship poses numerous risks to participants if there is a loss of privacy or confidentiality, such as immigration court proceedings. Additionally, asking legal status questions when building relationships may create an environment of surveillance instead of trust and rapport [8]. To maximize comfort and privacy, interviews occurred at a time and location convenient for participants and recorded after permission was given by the interviewee.

Our positionality – our social position in relation to the fishermen – was also important to identify and recognize. To moderate power dynamics, while acknowledging we could not fully alleviate them, we hired local, trained interpreters fluent in Vietnamese and Spanish for all interviews and observations. As academics who are neither of Vietnamese nor Latinx descent, it was important that the interpreters were part of the participant’s community with ties to the fishing industry. Although (im)migrant fishermen in the Gulf Coast have been deemed a ‘hard-to-reach population,’ our research found that they were instead an ‘under-reached population,’ meaning that they are accessible, but for a multiplicity of reasons (e.g., location of researchers or inability to speak their native language) they are underrepresented in research.

At the site where the health clinic was ultimately implemented, the study team met with shrimpers weekly for the first 6-months, and then monthly thereafter. Observational meetings were not recorded since they were focused on relationship-building, but field notes were taken. Flyers were distributed to managers and dock workers advertising the free health clinic and study team members continued to meet with shrimpers to identify needs for the clinic.

### Measures

Alongside our relationship-building initiatives, we also developed in-depth interview guides to facilitate rapport and trust with participants [9]. A standardized protocol was followed when developing the guides with the following components: (a) explanation of study, participant’s right to stop the interview at any time, and the study’s confidential nature; (b) use of recorders; (c) reason for taking notes; and (d) importance of not using any specific names. Further, a trusting and respectful environment, as described above, was created to maximize the likelihood that confidentiality was assured, and that participants felt comfortable talking freely and openly during the interviews. Following the interview, the interviewer documented any additional notes from the session [9]. Interview questions focused on past injuries, what safety and injury prevention mean to them, how they obtain health care services when injured, their perception of danger, and ended with shrimpers giving ideas for safety interventions.

### Data analysis

In this paper, we only discuss the results from the intervention that was identified by fishermen in the interviews, the fishermen’s health clinic; therefore, we limited data analysis to participation during the one-day event. Analysis included field notes from participant observation and tracking forms that documented each station’s participation rates.

## Results

### Interview findings

Findings from the interviews showed a general lack of health care options for the workers. Although the initial research question focused on injury prevention, fishermen discussed these issues within a larger structural and social context of having little to no health care. Their lack of health care options varied, but included lack of coverage, little time to make appointments between shrimping trips, financial and language barriers, and lost, missing, or incorrect citizenship documentation.

To address this finding, researchers at the University of Texas Medical Branch’s (UTMB) Center for Violence Prevention, along with other UTMB partners, implemented a free health clinic for (im)migrant workers on July 12, 2021 (3 days prior to the opening of the Texas Shrimp season) on the docks of a Texas Gulf Coast city. The services offered, described in detail below, came directly from the CBPR interviews with the fishermen.

### Intervention findings

Participants in the mobile clinic were seafood workers (i.e., shrimp fishermen and dock workers) and their wives and children. They were predominately male (~ 95%), identified as racial/ethnic minorities (~ 90% Vietnamese, ~ 10% Latinx), and were between the ages of 30-65, except for two children who were both under the age of 10. The Fishermen’s Health Clinic was planned over the span of 2 months, which included finding volunteers, ordering supplies, outreaching to shrimpers, and gaining access to the single-shot Johnson and Johnson COVID-19 vaccine; this latter coordination effort was necessary given the difficulty in arranging a second shot with a migratory population. To access the greatest number of workers with the highest likelihood of disparities, we implemented the health clinic in the morning and early afternoon (9 am – 1 pm). Implementing the intervention just prior to the opening of the Texas shrimp season provided nearly universal access to area shrimpers, including some of the most vulnerable workers who would be fishing during the roughest conditions (i.e., hurricane season).

**Fig. 1:**
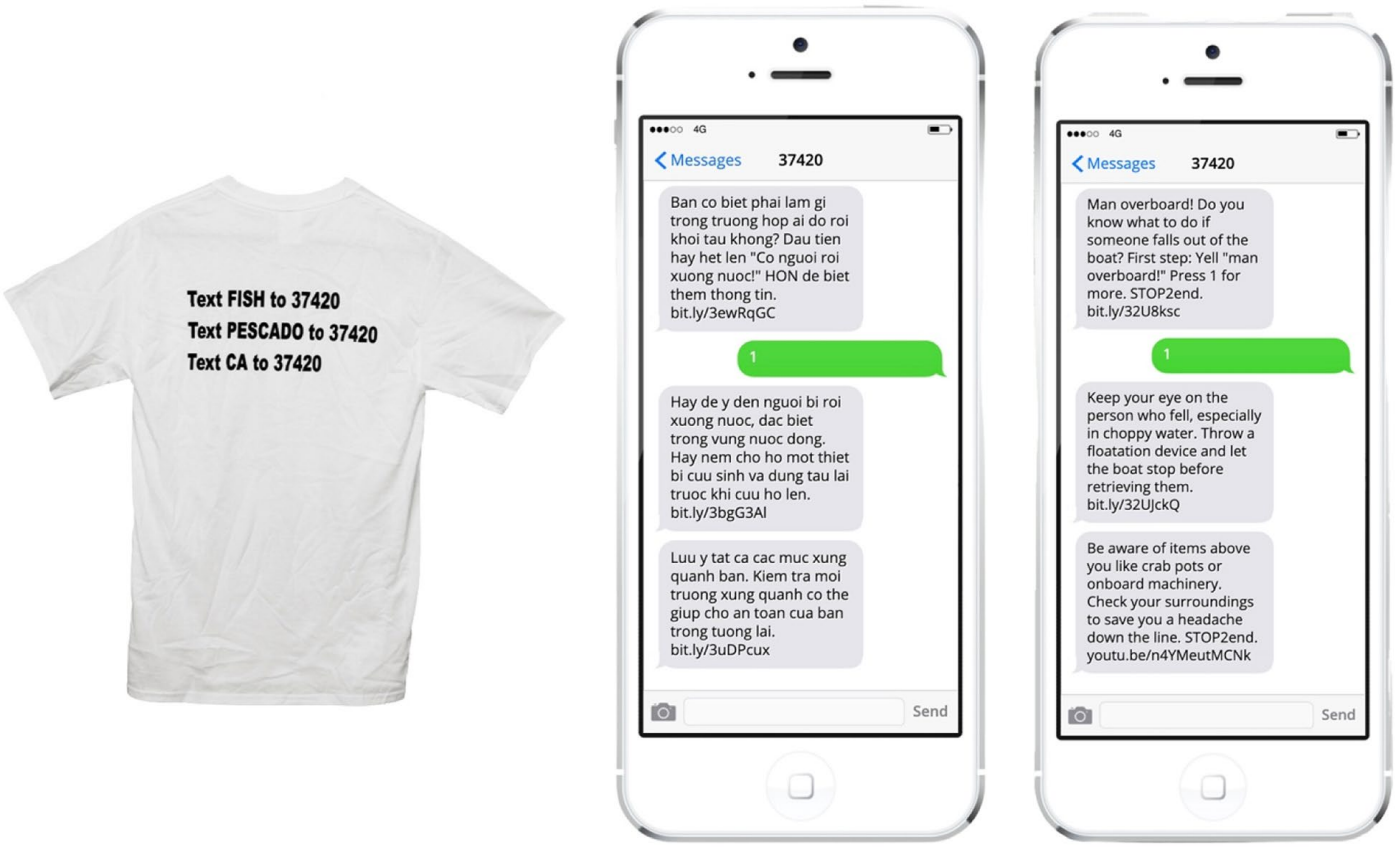
T-shirt with instructions on how to access the tri-lingual safety prevention texting service and example of texting campaign in Vietnamese and English

The clinic was staffed by volunteer faculty, staff, and students. In addition to vaccinating workers, we offered hypertension screening, diabetes screening including Point-of-Care Tests A1C, medical evaluation services by physicians and nurse practitioners, wound care supplies including appropriate antibiotics for marine skin infections, foot care, Occupational Therapy evaluation and treatment, boxed lunches, and medical kit bags, including an emergency medical guide (see Table 1). We also handed out t-shirts with instructions on how to access a safety prevention texting service (see Fig. 1), as well as structural and social service resources, including representatives who could assist with housing, food, toiletry, clothing, and transportation needs. Additionally, we were able to link participants to a full spectrum of free medical care services through an on-site physician and local free clinic.

**Table 1:** Items included in medical kits for the entire boat and for individuals

Medical Kit for entire boat (***n*** = 50)	Medical kit for each crew member (***n*** = 150)
Dry bag	Dry bag
Bandages	Hand sanitizer
Adhesive tape	Masks
Bandage strips and “butterfly” bandages in assorted sizes	Alcohol wipes
Rubber tourniquet or 16 French catheter	Tweezers
Nonstick sterile bandages and roller gauze	Toothbrush
2 in × 2 in Gauze	Floss
Large triangular bandage	Condoms
Disposable nonlatex examination gloves	Bandages
Tweezers	Muscle Rub
Thermometer	Wide brim hat
Liquid bandage	Paper fan
Trauma Kit with Quick Clot	Emergency Medical Guide
3 In Dressing	
4 in × 4 in Gauze Sterile PK2	
Trauma Pad	
Duct Tape	
Fracture/Sprain Bandage	
Triangular wound care	
Antiseptic wipe medical information	
Trauma and accident management instructions	
Hand wipes	
QuikClot Sport 25 g

The services offered were based on feedback from the fishermen about their most pertinent healthcare needs. However, some services that were requested could not be offered, such as eye and dental care. Although our team attempted to provide these services, faculty or residents could not be secured on the day of the clinic.

The highest number of visits were to the Medical Kit (*n* = 150), Boxed Lunches (*n* = 100), and Diabetes Screening (*n* = 40) stations. This was followed by visits to the Blood Pressure Screening (*n* = 32), Antibiotic Prescriptions (*n* = 17), COVID vaccine (n = 10), Foot Care (*n* = 8), and Occupational Therapy (*n* = 5) stations. At least one person was diagnosed with Type 2 diabetes, 5 were sent for diabetes tests, one person was immediately admitted to the hospital, and one person was diagnosed with high blood pressure and transported to a local free clinic. The latter person received free medication and a follow-up appointment with transportation options provided. Additionally, at least 5 other workers received follow-up appointments.

During the clinic, there were additional negotiations that needed to be resolved between the research/clinical team and fishermen/managers. When arriving on site, for instance, the parking lot was not cleared of cars, as discussed with management. Our team quickly rearranged clinical sites to be accessible to the fishermen receiving services and accommodate managers needing to transport shrimp, while also providing necessities to clinicians, such as power outlets and internet capability. These ‘in the moment’ negotiations could not have been possible without long-term trust and rapport built between the fishermen, managers, researchers, and clinicians. Our research team reminded clinical volunteers that we were guests on the docks and at the same time we reminded management of our goals for the day, thereby keeping peaceful relationships between the many different entities and personalities present.

Additionally, during the clinic a cultural miscommunication occurred between clinical volunteers and a fisherman, which highlights how the good intentions of volunteers can create tensions when not approached through a relationship of trust and rapport. A captain, who is one of our strongest supporters, began handing out medical kit bags to deck hands on the boat instead of encouraging the fishermen to approach the tent where the bags were stored. Clinical volunteers saw this happening, and not knowing his relationship to our team, asked that he stop taking the bags to them. The captain became verbally angry at what he took as an accusation of mishandling the medical kit bags and left the clinic. The research team was able to discuss the miscommunication with him, learning that he was giving the deck hands the medical kit bags as a sign of goodwill and to show them what was being offered. He explained that most deck hands, who did not speak English and may be undocumented, would not trust the many strangers at the docks unless offered something tangible. In turn, we apologized to him for the miscommunication and explained the situation to clinical volunteers. The captain ultimately returned to the clinic and continued assisting the fishermen.

## Discussion

As our findings show, the fishermen’s lack of health care options was rooted in structural and social contexts, such as financial, language, and documentation barriers. Work-related and nonwork-related injuries and illnesses were interconnected and influenced individual decision-making processes [3, 10–13]. Although work-related research, such as occupational health studies, have historical roots in social medicine they have involved into more technical and applied fields dedicated to identifying and eliminating workplace hazards without taking structural or social factors into account [3, 10, 14]. However, social determinants of health, such as housing stability and employment status impact exposure to work-related harm and need to be better understood.

Therefore, our clinic took aim at the varying structural and social issues that workers in low-income and dangerous environments face, while also prioritizing their needs and expertise. As discussed in the results, implementing the clinic at a convenient date, time, and location enhanced healthcare access to shrimp fishermen during the dock’s most populated time of the year. The study team, drawing from social determinants of health research and Maslow’s hierarchy of needs, ensured fishermen had basic physiological (e.g., water, food, sleep, shelter) and safety (e.g., basic healthcare, security) needs met through the health clinic before determining specific slips, trips, and falls interventions. Maslow’s hierarchy of needs is a well-recognized framework to understand, prioritize, and determine appropriate interventions [13]. Although not all needs could be met (i.e., eye or dental care), the team prioritized what fishermen said they needed with what was readily available.

The intervention, which focused on structural and social risk factors using CBPR approaches generated knowledge that was used to promote equity in the workplace and stimulated policies that bridged research and practice [11, 15, 16]. For instance, after the success of the first health clinic and identified needs among the community, our University helped us secure dedicated clinicians to work with our study team on connecting shrimpers to health and social services. The success of the primary (i.e., direct health and social needs met) and secondary (i.e., increase the likelihood of adopting future interventions) outcomes of the Fishermen’s Health Clinic now allows us to incorporate the model in subsequent efforts with this and other (im)migrant populations.

Finally, our study showed how relationship-building through CBPR helped mitigate tensions and ensured participants were recognized as equal partners in the intervention. Other studies have also shown how involving populations in the research process who have been made vulnerable by historical and current policies produces more relevant results [6, 17, 18]. Similarly, a systematic review of racial/ethnic minorities reported mistrust, competing demands, unintended outcomes, lack of access to information, stigma, health insurance, and legal status as shared barriers to health research participation [7]. Engaging the shrimp fishermen in the research process allowed us to gain a deeper understanding of the cultural issues and structural/social factors related to the adoption of safety interventions, and to create a more relevant intervention that respected them as experts in their own lives and healthcare needs.

## Conclusions

Following CBPR methods, the study team implemented a free health clinic that was centered on shrimp fishermen’s experiences and healthcare needs. Although CBPR or free health clinics are by no means novel in concept, these methods as applied to high-risk occupational settings with under-reached populations, such as (im)migrant workers, have enhanced the likelihood of adopting selected injury prevention measures [19]. This intervention adds to the growing literature around the benefits of using CBPR with populations, including (im)migrant workers specifically and the field of occupational health more broadly [10].

## Data Availability

The datasets used and analyzed during the current study are available from the corresponding author on reasonable request.

## Abbreviations

CBPR: Community based participatory research
CDC: Centers for Disease Control and Prevention
NIOSH: National Institute of Occupational Safety and Health
UTMB: University of Texas Medical Branch
